# Tumefactive Multiple Sclerosis, A Rare Variant Presenting as Multiple Ring-enhancing Lesions in an Immunocompetent Patient: A Case Report

**DOI:** 10.7759/cureus.3738

**Published:** 2018-12-17

**Authors:** Kamran Zaheer, Aman N Ajmeri, Monider Singh, Mohamed S Suliman, Samson Teka

**Affiliations:** 1 Internal Medicine, Joan C Edwards School of Medicine at Marshall University, Huntington, USA

**Keywords:** multiple sclerosis, immunomodulators, tms therapy, ms therapy, immunocompetent, ring-enhancing lesion, space-occupying lesion, tumefactive multiple sclerosis (tms)

## Abstract

Tumefactive multiple sclerosis (TMS) is a rare entity which can be difficult to diagnose unless definitive diagnostic measures are taken. TMS is characterized by solitary or multiple lesions that are sized > 2 cm, with/without mass effect, edema, and ring enhancement on magnetic resonance imaging (MRI). The demyelinating lesion can mimic infections, vascular lesions, and inflammatory lesions. The clinical presentation is highly dependent on the area of the brain which is affected, and this can lead to a variety of signs and symptoms. Herein, we present the case of a 40-year-old immunocompetent female with a history of right-sided numbness of her face, arm, and leg associated with muscle weakness for about a week. Workup included an MRI showing ring-enhancing lesions in the white matter of the brain, zero oligoclonal bands in the CSF, a normal immunoglobulin G (IgG) index, and an elevated myelin basic protein (MBP) in the CSF. A biopsy was obtained that showed predominant macrophage infiltrate with loss of myelin but the preservation of axons. Suspecting a demyelinating pathology, the patient was informed that she would be started on intravenous dexamethasone for an eight-day course. With subsequent completion of this course in the hospital, the patient was discharged on oral prednisone daily for a month and a referral leading to a definitive diagnosis of TMS. The patient was started on interferon beta-1a and subsequently relapsed due to noncompliance. However, further workup showed a reduction in the mass-like lesions and a response to therapy. If suspicion for TMS is high despite workup, steroids can be used with immunomodulators in the interim to combat symptoms and potentially reduce lesions and potentially subvert the need for biopsy.

## Introduction

Neurological symptoms require a physician to take into consideration many differentials ranging from vascular, metabolic, to infectious causes. Multiple sclerosis (MS) is a chronic inflammatory demyelinating disease known for relapsing and remitting episodes [1]. Tumefactive multiple sclerosis (TMS) is a rare variant that occurs in one per 1,000 cases of MS, or in three cases per million patients per year [2]. TMS is known for having varying clinical and radiologic features that resemble a neoplastic, infectious, or inflammatory process which can make for a challenging diagnosis without utilizing definite diagnostic measures [1, 3]. The clinical presentation is highly dependent on the area of the brain that is affected, and this can lead to a variety of signs and symptoms which include, but is not limited to, ﻿headache, cognitive abnormalities, mental confusion, aphasia, apraxia, and seizures [3].

TMS as an entity is a demyelinating disease, and it tends to present as a space-occupying lesion that has tumor-like effects [4]. Diagnostic imaging, in particular, is done with magnetic resonance and is confirmed on histology via biopsy [5-6]. Workup tends to include cerebrospinal fluid analysis to give the diagnostic yield more value as it tends to rule out other causes [4]. Literature has shown that it can present with ring enhancement on MRI with gadolinium, and on histopathology, it typically shows a combination of foamy macrophages and reactive astrocytes in the setting of perivascular inflammation with the absence of malignant cells [1, 5, 7-8].

While the prognosis is mostly benign in nature, the symptoms typically remit with doses of high-intensity steroids [9]. In addition to using steroids to help resolve acute symptoms, immunomodulators can help reduce the lesions present in some cases [9-10]. This was the case in our report; a young adult female had a full workup done for neurological symptoms which, in turn, led to the diagnosis and treatment for TMS.

## Case presentation

**Figure 1:**
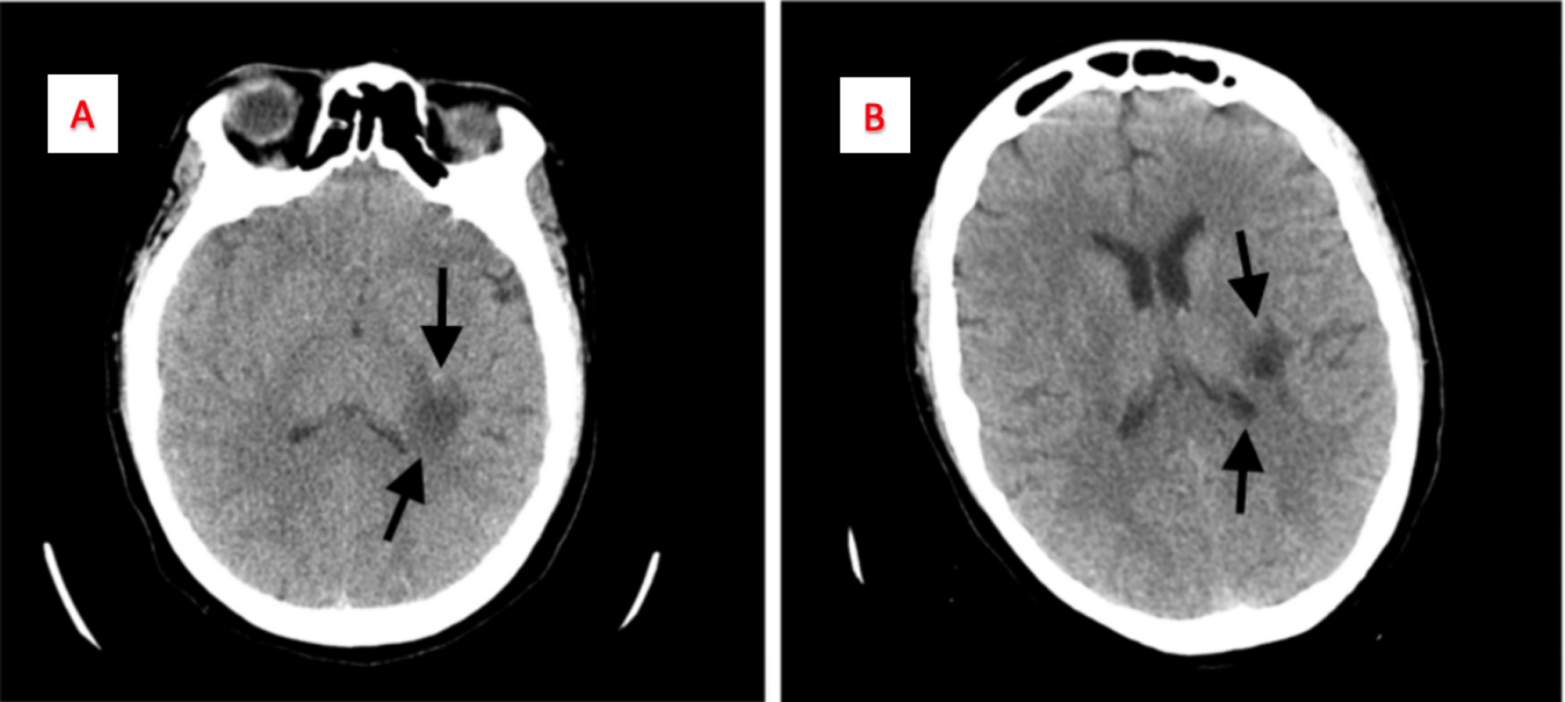
Computed tomography (CT) of the head without contrast Panel A: Displays a 2 x 1.5 cm hypodense lesion adjacent to the posterior basal ganglia/lateral thalamus. Panel B: Displays the same lesion at the level of the deep white matter adjacent to the posterior left lateral ventricle.

A 40-year-old Caucasian female presented to our facility with a five-day history of right-sided numbness. She was referred to us from a small area hospital with a concern for a mass following computed tomography (CT) of the head. The CT report showed a 2 x 1.5 cm hypodense lesion adjacent to the posterior left basal ganglia/lateral thalamus and deep white matter adjacent to the posterior left lateral ventricle (Figure 1). She complained of numbness of the right side of her face, right arm and leg, and a fall a couple of days prior to presentation. There was no numbness or weakness of the left side. She also complained of a headache and initially thought that her symptoms were related to it. The patient did not report any recent weight loss, fever, chills, or any other associated symptoms. Her past medical history was significant for migraine headache, hypothyroidism, chronic obstructive pulmonary disease (COPD), and lumbar radiculopathy with a herniated disc. Her past surgical history was significant for an L5-S1 microdiscectomy in 2013. Additional pertinent history included the patient having smoked a pack of cigarettes a day for 22 years and having a family history significant for lung cancer. The patient was hypertensive and all other vitals were stable. Physical examination revealed multiple neurologic findings, including decreased sensation to light touch on the right side of her face with the sensation of the tongue being intact, and decreased sensation in the right upper arm and right lateral leg below the knee. Decreased strength in her right upper and lower extremity (3/5 diffusely) was noted. A cardiopulmonary examination was unremarkable.

**Figure 2:**
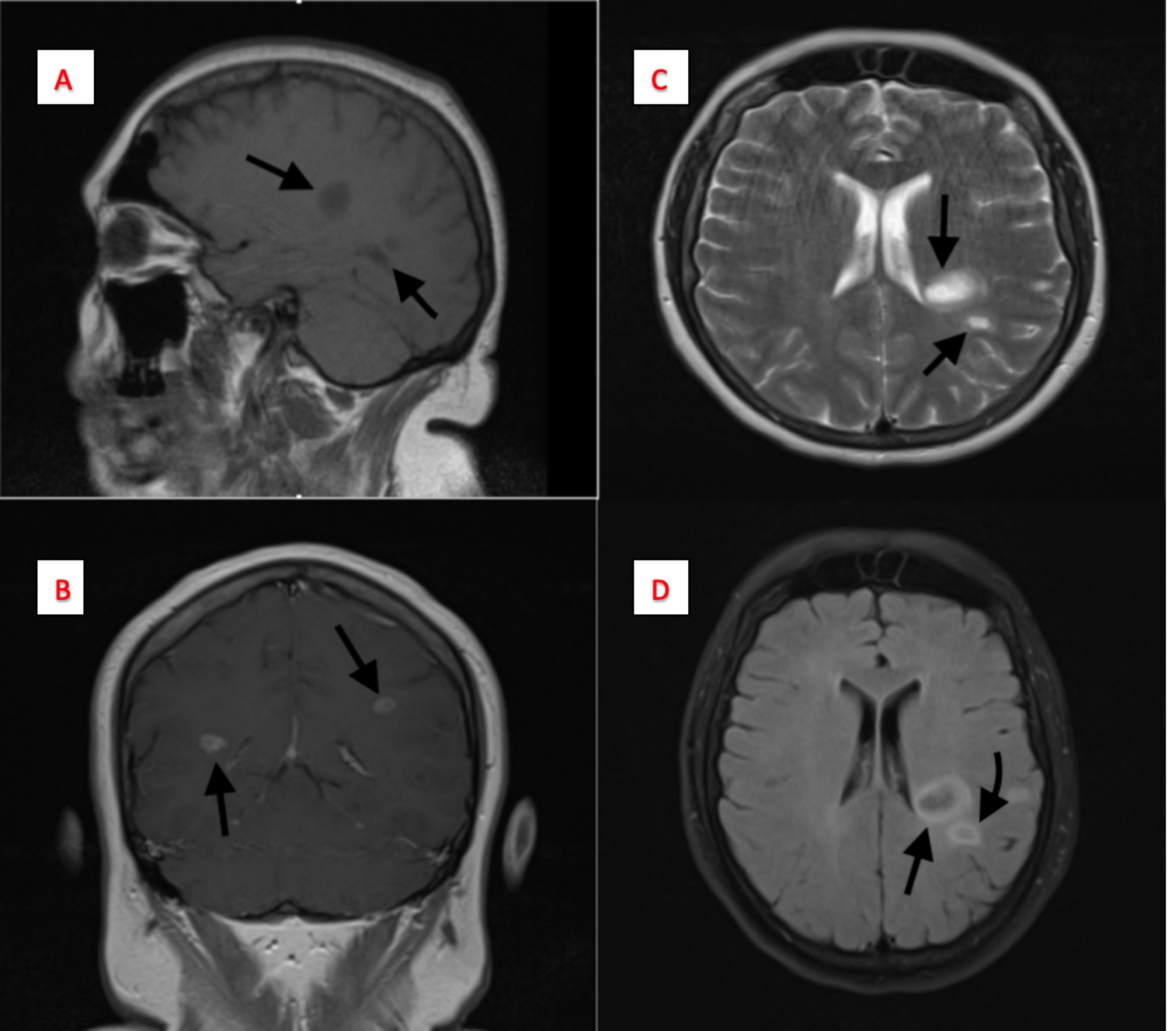
Magnetic resonance imaging (MRI) of the brain with/without contrast Panel A: Displays a sagittal T1 image with two hypodense lesions; Panel B: Displays a T1 contrast enhanced coronal image of lesions in both cerebral hemispheres; Panel C: Displays a T2-weighted image with two (16 x 22 mm and 7 x 12 mm) periatrial ring-enhancing lesions in the left periatrial white matter; Panel D: Displays flair image of the periventricular lesion.

MRI with gadolinium enhancement demonstrated multiple ring-enhancing lesions in the right and left periatrial white matter, the subcortical area of the left parietal lobe, and left temporal lobe on fluid-attenuated inversion recovery (FLAIR), T1-weighted (T1W), and T2-weighted (T2W) images (Figure 2). It was associated with varying degrees of central liquefactive necrosis and perilesional edema. This suggested a differential of metastatic, inflammatory, infectious, or demyelinating disease. Further workup for metastatic disease was initiated with modalities, including CT of the chest, abdomen, and pelvis without contrast, followed by a bone scan, and x-rays of all four extremities, which were all reported to be negative. After metastasis was ruled out, the primary focus was turned towards multiple sclerosis and infectious pathologies.

Workup for infectious causes was initiated with cerebrospinal fluid (CSF) analysis for aerobes and anaerobes, toxoplasma, Cryptococcus, fungal, and human immunodeficiency virus (HIV) infections. All studies proved to be negative. CSF workup for multiple sclerosis displayed zero oligoclonal bands. CSF concentration of myelin basic protein was elevated with a value of 7.1 (0.0 - 0.12 ng/ml), in addition to cytology results revealing mature lymphocytes. CSF protein and antibody analysis yielded insignificant results and included a normal IgG index. A serum IgG of 564 (700 - 1,600 mg/dL) and a CSF IgG being 1.4 (0.0 - 8.6 mg/dL) were obtained and were unremarkable. This further directed the need for a biopsy of the left thalamic lesion.

Histologic sections demonstrated a predominant macrophage infiltrate that was relatively demarcated from the surrounding white matter. The immunohistochemical stains performed at an advanced medical center demonstrated olig2 to label glia, which can be interpreted as promoted recovery. A myelin stain (e.g., Luxol fast blue) showed loss of myelin with relative preservation of the axons on neurofilament protein (sm31). Oncologic markers were negative. Findings were significant for a macrophage-rich lesion and suggestive of demyelinating disease.

After ruling out infectious and malignant conditions, we ultimately determined this to be an unknown inflammatory condition with a strong suspicion of multiple sclerosis (MS) at the forefront of possible etiologies. The patient was started on an eight-day course of intravenous dexamethasone, 4 mg every six to eight hours. The course of intravenous dexamethasone was completed in the hospital, and the patient was discharged to rehab with a month of per os (p.o.) prednisone, 20 mg daily, followed by a taper. She was also referred to a specialized medical center for MS confirmation, where they were better able to confirm the diagnosis as complex tumefactive multiple sclerosis (oligoclonal band negative). As a result, the patient was started on interferon beta-1a which was taken three times per week via subcutaneous injection.

**Figure 3:**
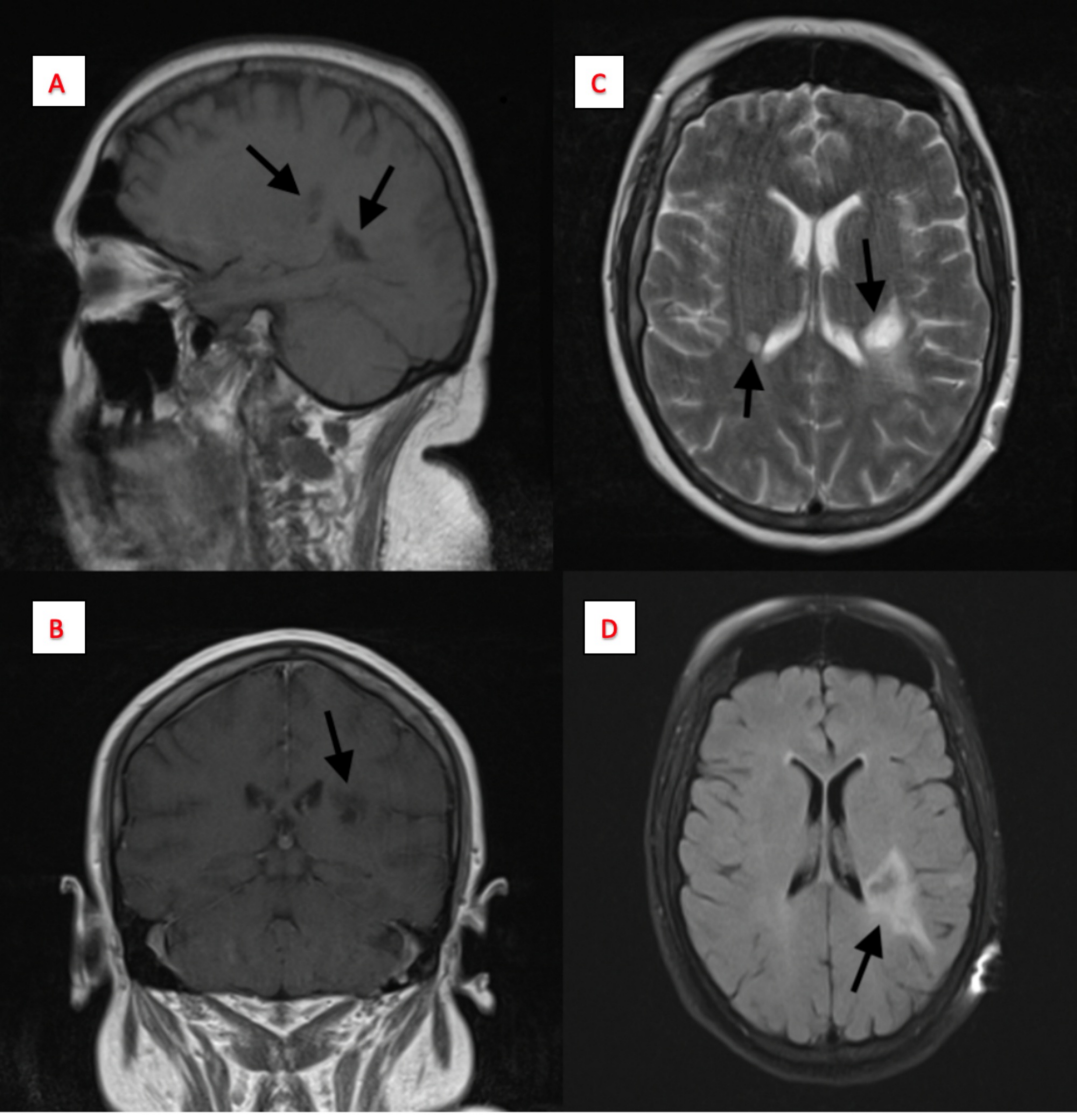
Magnetic resonance imaging (MRI) of the brain with/without contrast four months post-biopsy and treatment Panel A: Displays sagittal T1 image with two reduced hypodense lesions; Panel B: Displays a T1 contrast-enhanced coronal image of reduced lesions in both cerebral hemispheres; Panel C: Displays a T2-weighted image with two reduced periatrial ring-enhancing lesions in the left periatrial white matter; Panel D: Displays the fluid-attenuated inversion recovery (FLAIR) image of reduced periventricular lesion. Note: Tumor dimensions on this scan were not available.

The patient subsequently returned to our facility for a similar episode four months following the initial presentation but with the now confirmed diagnosis of TMS. This was assessed as a relapse due to noncompliance of weekly injections. On imaging, MRI showed a reduction in the mass-like lesions (Figure 3), leading us to believe the patient was responding to treatment. The patient was monitored for two days and discharged to rehab on oral teriflunomide.

## Discussion

Demyelinating pathologies of the central nervous system are regularly encountered, with MS being the most common [11]. Conventionally, it is considered to be an autoimmune disorder mediated by an abnormal T cell attack against the central nervous system (CNS) components, particularly myelin [10]. MS is a disease that is classically diagnosed with clinical and/or radiographic evidence of dissemination in time and space [3]. Typically, MS does not require biopsy for confirmation; however, the diagnosis of TMS can prove to be difficult because the clinical and radiologic findings can be indistinguishable from other brain lesions, including tumors, and may require biopsy [12]. Suggestive features for demyelinating lesions on histopathology include an abundance of foamy macrophages while lacking coagulative necrosis, evenly distributed plump, reactive astrocytes with closely intermingled macrophages, absences of microvascular proliferation, perivascular inflammation, and relative axonal preservation [12].

Before considering biopsy, imaging plays a crucial role in distinguishing cerebral lesions. MRI is considered the gold standard neuroimaging modality for a TMS diagnosis. Characteristically, these lesions are hyperintense, over 2 cm in size, and show ring enhancement with gadolinium [12-13]. The differential for ring-enhancing lesions on MRI includes a primary or metastatic neoplasm, pyogenic abscess, tuberculoma, neurocysticercosis, resolving hematoma, tumefactive demyelination, or radiation necrosis [14]. In patients with TMS, symptoms tend to vary depending on the area involved, with 50% presenting with motor deficits [3]. It can present with headache, cognitive abnormalities, mental confusion, apraxia, aphasia, motor deficits, and/or seizures. In our case, with the combination of the patient's neurological deficits and multiple ring-enhancing lesions, multiple imaging modalities led the workup to be diagnostically extensive and challenging.

Another diagnostic modality that can help differentiate neoplasms from TMS lesions is perfusion imaging. This modality measures the mean relative blood volume within the lesion. TMS lesions will show significantly less relative blood volume compared to neoplasms, but this may be inconclusive because of the variability in different types of tumors, especially in glioblastoma multiforme and primary CNS lymphoma. TMS lesions have contrast enhancement with gadolinium on MRI in 95% - 100% of patients with varying patterns, such as open or closed rings, diffuse, concentric, homogenous, or punctuate pattern. The ring enhancement in TMS lesions represents an advancing area of active inflammation away from the non-enhancing core, which is indicative of chronic inflammation [12].

Diagnostically, in addition to imaging, CSF analysis plays an important role to help distinguish TMS lesions from other pathologies. The main features typically include an elevation in the IgG index and oligoclonal bands (OCBs). Literature has illustrated that OCBs are present in 90% of patients with pre-established MS compared to 52% of patients who present with tumefactive demyelination as the first event [12]. With the frequency of positive findings in ancillary tests for TMS varying between cases, ours followed that general trend with CSF analysis showing zero OCBs and a normal IgG index.

According to a retrospective study by Altintas et al., treatment with high-dose IV steroids resulted in 82.7% of cases showing a significant response with regression of lesions in 76% of cases [13]. In our case, the patient responded to acute treatment with steroids, and although she had a relapse, she was showing clinical and radiological improvement of her pathology. Our report adds to the growing contingent of TMS cases. It is important for clinicians to recognize that with only 52% of patients having a positive ancillary test, TMS is not ruled out from the differential. A biopsy is ultimately diagnostic but is usually considered the last line when other imaging modalities are unclear. Perhaps multimodal imaging and response to high-dose steroids after the workup to rule out primary malignancy could have eliminated the need for a biopsy in our patient.

## Conclusions

TMS is a rare variant, and in our case, it showed that with unclear diagnostic imaging, a normal IgG index, and negative OCBs, the differential of TMS couldn’t be excluded. As a result, a biopsy to attain a histopathological diagnosis was required in this case. However, multimodal imaging approaches may help distinguish tumor presentations from their mimics and avoid biopsy in some cases. If clinical suspicion is high despite workup, steroids can be used with immunomodulators in the interim to combat symptoms, reduce lesions, and potentially subvert the need for biopsy.
